# Barriers to and enablers of Pakistani pharmaceutical export to regulated markets: regulatory perspective

**DOI:** 10.1080/20523211.2025.2564828

**Published:** 2025-11-28

**Authors:** Zobia Mubarak, Arslan Javed, Mobina Manzoor, Amna Riaz, Fozia Akbar, Hina Shahbaz, Farooq Bashir Butt, Gabriele De Rubis, Furqan Khurshid Hashmi, Nasir Abbas, Nadeem Irfan Bukhari

**Affiliations:** a Punjab University College of Pharmacy, University of Punjab, Lahore, Pakistan; b Primary and Secondary Healthcare Department, GOVT of the Punjab, Lahore, Pakistan; c Department of Pharmacy, Lahore College for Women University, Lahore, Pakistan; d The Women’s University, Multan, Pakistan; e Discipline of Pharmacy, Graduate School of Health, University of Technology Sydney, Ultimo, Australia; f Faculty of Pharmaceutical Sciences, Qarshi University, Lahore, Pakistan

**Keywords:** Drug regulatory authority of Pakistan, good manufacturing practices, pharmaceutical export, research and development, stringent regulatory authority

## Abstract

**Background:**

The pharmaceutical sector in Pakistan has grown over a period; however, there are certain barriers within the framework that regulate the growth and export of pharmaceuticals in the country. The purpose of this study was to highlight the current challenges for the pharmaceuticals’ export from Pakistan, especially to countries with stringent regulatory authorities (SRAs), from a regulatory perspective, and to identify the facilitators that may help resolve these challenges.

**Methods:**

In this qualitative study, data were collected from the participants from the regional offices of Drug Regulatory Authority of Pakistan (DRAP), located in Lahore, Islamabad, and Quetta. Regulatory participants with a minimum experience of 6 years and the designation of an assistant director or above were recruited through purposive sampling. Semi-structured interviews were used to collect information from regulatory experts. Inductive thematic content analysis was employed to conclude the data.

Data analysis generated 5 themes and 20 categories/codes. Poor export performance and pharmaceutical growth was attributed to barriers such as: inadequate industrial research and development, non-compliance with the current standards of good manufacturing practices (cGMP), absence of regulatory requirements for high-tech equipment, insufficient academia-industry collaboration, shortage of locally manufactured active pharmaceutical ingredients and lack of support from the government. Accreditation with international organisations such as the World Health Organisation and the Pharmaceutical Inspection Co-operation Scheme was considered deficient. DRAP, in coordination with the Trade Development Authority, could enhance pharmaceutical exports. Addressing the above challenges could boost the export and expand international market share of Pakistani Pharmaceuticals in countries with SRAs.

**Conclusion:**

The cGMP compliance, regulatory assistance, and appropriate research and development, including bioequivalence studies, could contribute to export enhancement to SRA countries. Further, the exports from Pakistan to countries with SRAs could be enhanced with DRAP's coordination with the trade development authority of Pakistan.

## 1. Background

Pakistan's pharmaceutical sector is steadily growing, and currently, there are 759 pharmaceutical manufacturing units, inclusive of 29 multinational companies (DRAP, 2022a). The annual sale of the domestic pharmaceutical sector is 3.1 billion USD, which mainly includes systemic anti-infectives, followed by the drugs for gastrointestinal and metabolic disorders as the major categories of sold finished pharmaceuticals (PPMA, 2021). Since, pharmaceutical sector is highly regulated, Pakistan has also adopted the trend of a drug regulatory authority, named as the Drug Regulatory Authority of Pakistan (DRAP) under the Ministry of National Health Services since 2012 (Rashid, 2015) for the enforcement of the DRAP Act 1976 (DRAP, 2012). This Act pertains to drug regulation, licensing, manufacturing, registration, pricing, import, and export (Atif et al., 2017) through the technical directorates of DRAP (DRAP, 2012). Domestic pharmaceutical manufacturers lack the mandatory international standards and certifications that are required for export of pharmaceuticals to countries with strict regulatory authorities (SRAs). Thus, pharmaceutical export has primarily been confined to the underdeveloped countries or those that have semi-regulated pharmaceutical markets. The current export destinations for the pharmaceutical export are mainly Central and Southeast Asia, Africa, the Far East, and South America. Nevertheless, export to this segment is minor and has little diversification or expansion over the last fifteen years (Jibran, 2015). A few national pharmaceutical companies have been qualified to submit product dossiers for export to the Philippines, a semi-regulated market. The domestic pharmaceutical industry could not target Middle Eastern countries, though they are semi-regulated markets. Afghanistan, being an unregulated market, has been consistently the largest export destination for Pakistan, nevertheless, with a worth of 67 million USD in 2019. Many companies are located near the Afghan border in Peshawar and have focused their production and aiding other firms in exporting products to Afghanistan (PRIME, 2017).

In 2011, the pharmaceutical industry in Pakistan was reported to be of worth 1.64 billion USD and with an annual growth rate of 11% in 2019. Pharmaceutical was the 22nd largest export sector in Pakistan, with a global export share of merely 0.4% and a world ranking of 62, lower than other export categories such as rice, leather goods, fish, fruits and nuts, and minerals. Over the past five years, despite a 6% sector growth rate worldwide, Pakistan failed to increase its world export share due to a 0% growth rate in the domestic industry (Khan et al., 2021).

The government of Pakistan has already taken certain measures to increase the export of pharmaceuticals, among which the most significant is the constitution of a pharmaceutical export promotion committee, under the Ministry of National Health Services, Regulations and Control (Rashid, 2015). The tasks of the committee are to: a) assist the production of pharmaceuticals, b) review the progress of the export of pharmaceutical products, c) suggest achieving growth targets, d) make policy recommendations on export boost, e) find the problems faced by the exporters of pharmaceuticals, f) highlight the procedural bottlenecks, g) recommend redressals to eliminate issues at every level, and h) prepare the short-, intermediate-, and long-term plans of action. Nevertheless, the working pattern and the progress of this committee are not clear (News, 2019).

The reported key constraints, preventing Pakistani firms from exporting to SRA and developed countries, are many (Khan et al., 2021; Koli, 2010). Pharmaceutical exporters have to meet the increasingly strict quality, legal, and ethical standards for compliant manufacture. Global pharmaceutical regulators such as the Food and Drug Administration (FDA) of the United States, the Medicines and Healthcare Regulatory Authority (MHRA) of the United Kingdom, the Therapeutic Goods Administration (TGA) of Australia, and the Medicine Control Council (MCC) of South Africa continue to upgrade their standards (Koli, 2010). The safety and quality standards in the export destinations, excessive documentation, and limited local capacity for testing and bioequivalence (BE) studies are the other constraints (Khan et al., 2021). In the USA, a stringent regulatory country, the FDA requires the registration of foreign drug manufacturers and ensures BE of generic products, to the innovator brands, to be sold in the USA. Contrarily, not a single local manufacturing unit is FDA-registered, nor does an internationally recognised facility exist in Pakistan to provide FDA certification for export. The same applies to other stringent markets such as the EU, Japan, Australia, and Russia (Khan et al., 2021). Further, DRAP export rules require only a cGMP certificate with a valid drug registration for the issuance of an export certificate (DRAP, 2022b). BE of generics to the innovator brands is not a requirement for the local drug registration (DRAP, 2022c), another barrier reported as a hurdle in the export of generics from Pakistan (Hasan, 2012).

To the best of our knowledge, literature specifically targeting the issue related to pharmaceutical export from Pakistan is scarce. A few of the citations on the subject for the reluctance of the pharmaceutical industry for BE studies, and lack of or loose regulatory BE requirement (Bilal et al., 2016; Kemal, 2002), could not be considered as the regulatory perspectives. The response of the pharmaceutical industry, DRAP and the Government to the above remained unveiled in these studies. Further, the previous investigations have been carried out based on existing literature or mainly focusing on the industrial owners who were the members of the Pakistan Pharmaceutical Manufacturing Association (PPMA) (Ahmed & Chandani, 2020; Pakistan Today, 2013). Furthermore, the focus of the earlier reports was on the barriers faced by or exploring the potential existing in the national pharmaceutical industry (TDAP, 2022). The hurdles faced by the domestic pharmaceutical industry, with perspectives of the regulators and the reasons for the industrial noncompliance with the requirements of the SRA countries, remained unattended. Moreover, our previous study on the perception of pharmacy academia (Mubarak et al., 2024a), as one of the stakeholders on the same issue, also prompted us to assess the regulators’ perception of the issue under consideration. Henceforth, the current study uniquely and broadly highlighted the issues exclusively in drug export faced by the local pharmaceutical industry, as perceived by the DRAP professionals, regarded herein as regulators, and the areas where the domestic pharmaceutical industry needs improvement, as perceived by them. This study also proposed measures to deal with the above issues and the growth of pharmaceutical exports from Pakistan to SRA countries. Targeting SRA countries is expected to not only boost exports but also enhance the sustainability of the local pharmaceutical industry.

## 2. Methods

### 2.1. Study setting

The study was conducted in regional DRAP offices in Lahore, Quetta, and Islamabad. Following individual appointments, face-to-face interviews were conducted individually, while in the remote cities, the interviews were conducted through WhatsApp and video Zoom calls.

### 2.2. Study design

A phenomenology-based qualitative exploratory design, which encompasses the essence of the shared experience of the phenomenon, was employed in this study (Wilding & Whiteford, 2005). Such a qualitative method allows for flexibility in the design of research and in-depth insight into the respondent's attitude, perception, and experience. (Kitzinger, 1995). Qualitative methods also produce diverse thoughts and perspectives that respondents have about certain matters and reveal differing viewpoints (Berg & Lune, 2009). Additionally, in areas of research not extensively studied, the qualitative methods strive to uncover information that surveys may overlook (Dolan Mullen & Reynolds, 1978). Therefore, qualitative interviews were deemed to be the optimal selection for this research, aligning with its objectives and facilitating inductive approaches to generate concepts and hypotheses with greater potential for further investigation than other models (Entwistle et al., 1998).

#### 2.2.1. Study tool – interview schema

A semi-structured interview guide, focusing on questions that address the research problems and gaps in the literature, was developed based on an in-depth literature review and current prevailing practices in the study setting (Krska & Veitch, 2001). The services of a senior officer from the Drug Regulatory Authority were utilised to develop the interview guide (Fatima et al., 2019), which was subjected to cumulative and argumentative validation (Bittmann & Thomas, 2013). Later, the reliability of the interview guide was pilot-tested for thoroughness and relevance to the problem stated by two respondents from the relevant field. Nevertheless, these results were not included in the final study because some slight changes were suggested in the above. The final interview schema (see Supplemental Material), comprising a total of eighteen questions with additional sub-questions or probing questions, was made available for the real-time data collection.

#### 2.2.2. Study participants and inclusion criteria

The study samples comprised regulatory experts from both provincial and federal regulatory authorities, having pharmacy degree, a minimum of 6 years of experience, and working in the position of assistant director or above. Though a 3-year experience is a prerequisite for the position of Assistant Director in DRAP, a regulator with 6 years of working in different fields and sections of DRAP is expected to gain sufficient experience to understand the pharmaceutical policy and practice. The participants were expected to provide a neutral perspective on the questions asked.

#### 2.2.3. Sampling methods

A mixed sampling approach, purposive, convenience, and snowball, was used in this study. Initially, the purposive sampling method targeted the acquainted regulatory experts, meeting the inclusion criteria, based on preconceived ideas about the required characteristics of the sample. Since most of the regulatory experts were difficult to reach due to their busy schedules, therefore, convenience sampling was deemed appropriate for recruiting the participants who were easier to find and also consented to participate, verbally at this point, and later sent it through surface and email, and WhatsApp, where the interview was not face-to-face. The convenience sampling was followed by a snowball sampling strategy, utilising recommendations from the existing study participants who assisted in recruiting the additional participants.

#### 2.2.4. Sample size

The sample size was determined by applying the Morse saturation point criteria (Morse, 2000). When an apparent saturation point criterion was achieved, two additional interviews were conducted to confirm the above, in line with the literature (Mubarak et al., 2024a).

### 2.3. Data collection

Data were collected from 6th March to 30th April 2023 by the first author, who had previously completed her MPhil in Pharmacy and was well-trained to carry out this study. The language during the interview was English, as the participants could understand without difficulty on account of the use of this medium of instruction at the secondary and tertiary levels in Pakistan. Participants were encouraged to express their views and comments freely. However, the modification in response to an early question, after posing the next one, was not entertained. Nevertheless, to clarify the ambiguous response, probing questions were asked (Mubarak et al., 2024b). All interviews were audio-recorded, and the principal researcher (first author) took additional field notes.

### 2.4. Data analysis

The data was analyzed using inductive thematic content analysis (TCA). All the interviews were transcribed verbatim by the first author. Together, these insights have been described to help explain the main areas where the domestic pharmaceutical industry is lacking as compared to the developed countries with SRAs, and that were required to be improved to facilitate export from Pakistan and for business growth. The analytical method followed the six steps for TCA described earlier (Braun & Clarke, 2006). The first author transcribed all the audio (in English) on paper to familiarise herself with the data findings and generated the initial notes. A code was assigned to a single sentence, several sentences, or larger segments, summarising the actual sense of a phenomenon described by the interviewees that was relevant to answering the research questions. The data were cross-checked by other researchers to ensure credibility and trustworthiness (Lincoln & Guba, 1985). The list of codes was organised in MS Word to help identify the connections among them. Codes describing similar phenomena were grouped under one theme. Finally, the main findings were presented as the identified themes. The emerging themes and contents were then verified by the co-workers of the study. In qualitative research, utilising data saturation ensures continuation of data collection until no new themes emerge, thus it helps minimise the potential for researcher bias and strengthen the validity of the findings (Fusch & Ness, 2015; Johnson et al., 2020).

### 2.5. Results

A total of 15 regulatory experts were contacted, while 13 experts (86.7% response rate) consented to participate. The remaining 2 refused to participate due to their busy schedules. The saturation was reached, apparently at the 10th interview, but 2 additional interviews were conducted to confirm saturation as reported (Morse, 2000), leaving the 13th interview due to reaching saturation. Demographic details of the study participants are given in Table 1. Out of 12 participants, 8 were males and 4 were females. All of the regulatory experts hold an MPhil in Pharmacy, and 2 with additional master's in business administration (MBA), mostly working as the assistant director or above with experience of ≥ 6 years. Two participants were from the provincial regulatory department of Punjab, and the remaining 10 were from the Federal Government. The age of the respondents varied from 30 to 65 years. Each interview lasted for 30 to 40 min (Average 35 min).

**Table 1.** Demographic details of the participants (n = 12).

Characteristics	Category	Frequency	Percent
Age	30-35	4	33.3
	36-45	5	41.7
	> 45	3	25.0
Gender	Male	8	66.7
	Female	4	33.3
Educational status	M.Phil Pharmacy	10	83.3
	M.Phil, MBA	2	16.7
Experience (years)	6–10years	5	41.7
	11–15 years	5	41.7
	>15 years	2	16.6
Location	Lahore	6	50.0
	Islamabad	4	33.3
	Quetta	2	16.7
Designation	Assistant Director DRAP	6	50.0
	Deputy directors of DRAP	4	33.3
	Director of Operations (PQCB)	1	8.3
	Chief Drugs Controller (Punjab)	1	8.3

### 2.6. Thematic content analysis

The thematic analysis of the acquired data yielded 5 themes with 20 categories, representing the perspectives of the regulatory experts about the current status of local pharmaceuticals, barriers, and facilitators to pharmaceutical export from Pakistan. The emerging themes, categories/codes, and supporting quotations are highlighted in Table 2 and described in the preceding text.

**Table 2.** Themes, categories/ and supporting quotations (R = Regulator).

Themes	Categories/Codes	Response/Respondents	Selected supporting quotes
Comparison of the local industry with that in the developed countries	Positive/negative feedback	Comparable (Regulators: R1, R6, R7, R10 and R11)	R1: ‘Only national companies approved by international organizations, such as WHO, PICS, and Health Canada, are comparable to those present in the developed countries’. R6: ‘The entire national industry is not less than any developed country’. R10: ‘Quality of the national pharmaceutical industry is comparable to that of the international industry’. R11: ‘National industry is comparable, progressing, and will be more developed in the future’.
		Noncomparable (Regulators: R2, R3, R4, R5, R9, R8 and R12)	R2: ‘The national pharmaceutical industry needs improved implementation of the existing regulatory laws. R4: ‘Local pharmaceutical industry is mostly manufacturing generics, contrary to the developed countries, which have well-developed patent laws and intellectual property rights protection’. R12: ‘There are different categories of pharmaceutical companies, such as low, middle, and high levels; the latter two are comparable to the developed countries’.
Regulatory requirements in Pakistan and their impact on export	Requirement for minimum investment	Yes (Regulators: R1, R3, R4, R8, R9 and R12)	R1, R4, and R12: ‘There must be a minimum cap on investment for a manufacturing unit to implement the standards required for quality products and procedures’. R3: ‘The inter-collaborative projects of the pharmaceutical industry with higher investments will help boost exports’.
		No (Regulators: R2, R5, R6, R7, R10 and R11)	R7: ‘Rather than imposing a minimum limit on investment, the minimum standards should be followed’. R10: ‘There is no need for a minimum investment limitation, as a single product could be launched and exported by a small company for ease of doing business’.
	Requirements for the installation of high-tech pieces of equipment	Yes (Regulators: R4, R,5, R6, R8, R9 and R12)	R4: ‘The requirement for installation of high-tech equipment should be imposed as the big industries have high-tech instruments with data traceability, while the small units do not have the latest technologies’. R5 and R9: ‘cGMP essentially requires the latest and high-tech equipment and the high-tech facilities, including heating, ventilation, and air conditioning systems for every pharmaceutical unit’. R8: ‘The high-tech machinery should be installed after qualification and approval from the regulatory body’. R12: ‘The requirements for testing facility of product quality are ever-changing and keep on improving with the adoption of pharmacopeial methods, which will then become the compulsory regulatory requirement’.
		No (Regulators: R1, R2, R3, R7, R10, and R11)	R1 and R7: ‘Rather than the requirement for installing high-tech equipment, the industry should have enough resources for testing and producing the products with the desired quality’. R2: ‘High-tech equipment should be dependent on the products to be manufactured’. R3: ‘Currently, there exists a regulatory requirement on the equipment for necessary quality testing rather than the high-tech manufacturing instruments.’
	The requirement to apply for the export of the same product after local registration	No (Regulators: R1, R7 and R12)	R1, R4, R7, and R12: ‘No such regulatory requirement exists to apply for reregistration of a product for export after its local licensing and registration’. R4: ‘Such a requirement depends on firms; regulators facilitate registration for export’. R7: ‘Only local registration is applied by the manufacturer. DRAP cannot compel firms to export. However, the export-only registrations are granted by DRAP’. R12: ‘Export product reregistration is not mandatory; every industry has its own capacity. Hurdles are the investment and time-consuming dossier approval from DRAP’.
Incentives for companies that intend to export	Incentive or relaxation for export firms.	Yes (Regulators: R1, R3, R4, R6, R7, R8 and R9)	R1, R6: ‘An incentive of considering the future drug application on a fast-track basis exists’. R8: ‘There is the incentive of priority registration by the export regulations bureau’.
		No (Regulators: R2, R5, R10, R11, R12, R13, R14, R15)	R2, R5, R11, and R12: ‘There is no such incentive’. R11: ‘Regarding documentation, some waivers are given’. R12: ‘FIFO is applied in the DRAP registration process.
	Incentives regarding price or profit margins	No (Regulators: R1, R4, R6, R12)	R1: ‘There are no such incentives’. R4: No incentives of price or profit margins or brand preferences for an exporting firm, all companies are treated in the same way’. R6: ‘The drug price is fixed in Pakistan, while for export, the manufacturer is free to choose the price’. R12: ‘There is no such incentive in terms of the product price; however, facilitation is given in the case of product export’.
	Incentives for bioequivalence studies	No (Respondents: R1-R12)	R1: ‘There is no incentive for bioequivalence studies’. R3: ‘The incentive is not from DRAP. However, BE studies could be used as a marketing strategy by the manufacturers who intend to sell and export their products’. R4: ‘No incentive, but BE testing could benefit the industry for export’. R7 ‘Though a couple of centers have been started in Pakistan, there is no incentive or facilitation for such studies from DRAP. No BE studies are being performed locally. DRAP should technically support industry for these studies’.
Areas of improvement in the local industry (Barriers)	Research and development	R1 to R12	R1: ‘R&D is the backbone of the pharmaceutical industry, yet it is not well-developed in Pakistan and other developing countries’. R4: ‘Companies should have an active R&D, which should be working in collaboration with academia’. R7: ‘R&D is the backbone of any company, but the national pharmaceutical industry is not inclined to carry out such activity, and they apply only the cut-and-paste method’. R12: ‘The R&D sections are not involved in the development of new drug molecules and new precursors. No raw materials are being produced in the country’.
	Academia-industry linkage (AIL)	R1 to R12	R1: ‘Academia plays a significant role in research on the time-tested drugs. Globally, academic research plays an integral role in providing new drugs, and new indications for drugs’. R7: ‘A coordination between the domestic manufacturing units and the academic institutes is vital and pivotal. Research should be of an applied type’. R10: ‘The DRAP's Division of Pharmacy Services can facilitate in establishing the AILs’. R11: ‘Academia should get involved with the skilled human resources from the industry and regulatory bodies in teaching, for which at least half of faculty should be technical persons. DRAP and the Pharmacy Council of Pakistan should put a check on the above’. R12: ‘DRAP can bridge academia and industry and highlight the areas of improvement that can be achieved with AIL’.
	Utilization of the Central Research Fund (CRF)	R1 to R12	R1: ‘CRF can be utilized in developing the BE, clinical trials, and research centers for new molecules, and the provision of traceable working standards”. R4: “The CRF could be used for carrying out research, and capacity building of DRAP offices”. R6: “DRAP should start manufacturing of at least 10 to 20 of the most common raw materials utilizing CRF. The vaccine sector should be improved in the country’. R8: ‘With the collaboration of academia, CRF should be given to the research scholars. Moreover, a research Centre can also be established at the national level with the funds’. R10: ‘CRF could be spent on the improvement of both the research intellects and the regulatory system’. R11: ‘With CRF, the regulatory officers should be trained by regulators in the SRA countries; a platform should be provided for the manufacturers and regulatory authorities for promoting the strengths of the firms and improving the weaknesses in terms of new technologies’.
Ways to improve export (Facilitators)	Export start-up programme with regulatory assistance	R1 to R12	R1: ‘DRAP can play a role by providing information to the industry for target markets and expediting the drug registration and licensing’. R4: ‘DRAP should coordinate with the trade development authority of Pakistan with a joint vision to launch the idea of export of generics”. R6: “Start-up export programs should be examined, and guidance should be sought from DRAP on compliance with the required standards’. R 10: ‘DRAP should provide a platform for export, which so far is not available’.
	Compliance with cGMPs	R1 to R12	R2 and R4: ‘Potential of pharmaceuticals export exists, which can be tapped by complying with and uplifting the cGMP standards and instituting accreditations with the international bodies’. R3: ‘There is the potential of compliance with cGMP, and export could be improved by adhering to cGMPs and producing good-quality products’.
	Providing the local API manufacturing facility	R1 to R12	R1: ‘The potential of API manufacturing exists, and if they are produced domestically, then export could be increased’. R6: ‘The export potential is there; however, the API industry is lacking in Pakistan due to higher production costs. By providing benefits to the pharmaceutical API industry, exports can be enhanced’.
	Initiation of positive marketing and media campaigns	R1 to R12	R1: ‘The export potential is there, and exports can be enhanced after gaining trust in the international market by providing quality products and improving marketing standards in target countries’. R8: ‘The export potential is not being handled appropriately. Positive media campaigns and image building of industries can enhance export’. R12: ‘The existing export potential can be materialized by providing the industry opportunities in the international market through interactions and with knowledge and support from a regulatory authority’.
	Training of the DRAP officers and industrial staff	R1 to R12	R3: ‘DRAP should provide awareness to industrialists, collaborate and arrange meetings with the foreign regulators for gaining knowledge’. R6: ‘The training being provided currently is not sufficient. The industry should be trained frequently’. R2: The cGMP guidelines are there, but the knowledge of the drug inspectors is less; staff training should be arranged internationally’.
	Implementation of laws and standards by DRAP	R1 to R12	R1: ‘Legislation is there, yet its implementation is necessary’. R10: ‘Yes, business-friendly local legislation should be made’.
	Improving DRAP accreditation, certification, and procedures	R1 to R12	R1: ‘WHO and PICS certification of DRAP is going to help in targeting export to SRA countries for which DRAP is already attempting’. R8: ‘DRAP is in the process of achieving WHO and PICS memberships, which will boost the local pharmaceutical industry’. R6: ‘DRAP can reduce the time of approval, and a fast-track approval of applications for drug product registration should be implemented. It should help in documentation’. R 7: ‘DRAP should give registration of internationally accepted salts. There are so many salts registered in the world but not in Pakistan, for instance, erythropoietin and monoclonal antibodies’.
	Government support	R1 to R12	R9: ‘Tax and customs duty should be minimized on the import of raw materials and other items used in pharmaceuticals’. R10: ‘The Government should set a plan for the effective utilization of CRF. Provinces should be involved in the disbursement of this fund, and an R&D committee should be formed for effective utilization of this fund’. R11: ‘Subsidize the rates of electricity and labor payment to reduce the production cost’. R12: ‘Government should build the capacity of DRAP in terms of human resources for effective delivery of their task. Currently, the regulatory officers are in short supply, so they are overburdened.

#### 2.6.1. Theme 1: comparison of the local and developed countries’ pharmaceutical industry

Out of 12, 6 regulators believed that some national pharmaceutical firms have been approved by renowned international organisations like the World Health Organisation (WHO), Pharmaceutical Inspection Co-operation Scheme (PICS), the Ministry of Health and the Regulatory Authority (MHRA) UK, and Health Canada. Therefore, these units meet the international standards and could be considered on par with their global counterparts. Furthermore, some domestic companies are developing and progressing to achieve international certifications, demonstrating their commitment to quality and improvement. Thus, the domestic pharmaceutical industry was not perceived as inferior to that of the developed countries, exhibited the potential to excel in the future, and was significantly striving to achieve international recognition. While 3 regulators (R2, R5, and R6) believed that the local industry needs improvement in the cGMP compliance and regulations. Two respondents (R9 and R11) also classified the local companies at three levels: high, middle, and low, and opined that a general comparison between the industry in Pakistan and developed countries could not be possible, though the latter two categories could be compared. Hence, neither all respondents were positive nor negative in comparison between local and developed countries’ industry, showing a mixed status of industry in Pakistan, from the regulators’ perspectives.

#### 2.6.2. Theme 2: regulatory requirements in Pakistan and their impact on export

Questions were asked on the regulatory requirements to start a pharmaceutical business in Pakistan, which included minimum investment, installation of high-tech equipment, and the requirement to apply for the export of locally registered products. Regarding implementing minimum investment, 4 out of 12 respondents considered that imposing a limit of minimum investment for kicking off a pharmaceutical business is necessary to meet the quality standards and sustain and grow the business. Contrarily, 6 respondents favoured imposing the minimum standards, rather than capping a minimum investment for the national industry to produce high-quality products for enhanced export. Related to the installation of the latest high-tech instruments, 7 respondents indicated that cGMPs required so, but it was not a legislative requirement. Three respondents stated that high-tech equipment is a regulatory demand, while 2 opined that DRAP required installation only of the necessary equipment rather than the high-tech equipment. Three respondents stated the difference between big and small industries; the former have high-tech instruments with data traceability, while the latter do not have such facilities. A polarisation among the respondents on the issue was reflected, suggesting that the regulatory officers themselves were not fully familiar with the requirements, which might cause a gap in compliance with cGMP guidelines. While talking about the requirement of DRAP to apply for export, it was expressed that DRAP did not, nor possible to bind any company for a minimum investment. The minimum investment is greatly affected by the number of products a firm intends to launch – the launch of a single product requires less money. A criterion of minimum investment, if required to be implemented, should be for a viable business.

#### 2.6.3. Theme 3: incentives for companies that intend to export

A question on incentives for export-intending or exporting companies was asked in different ways. Regarding export facilitation, a blended response was received. Six out of 12 respondents claimed no incentive for exporting companies, while 6 said otherwise, narrating a priority registration of one molecule as an incentive for the firms exporting their product in a certain amount. For the grant of a special price or profit margin on a product for exporting firms, a unanimous opinion was against the existence of such an incentive, though companies could set their price for export products. Another question was related to any incentives for firms that want to get BE studies for their products. All regulators agreed upon the lack of any incentive for such a purpose, as it is not a mandatory regulatory requirement for local registration. A company, however, needs BE studies on its own if it intends to export its products to a country with an SRA.

#### 2.6.4. Theme 4: areas of improvement in the local industry (Barriers)

Limited R&D in the local pharmaceutical industry was reported as a prime issue by almost all the regulators. Mostly, generics are being manufactured without even the stability and other parameters assessment, and no research on the new molecules was conducted. All regulators unanimously highlighted the non-existent AIL as another barrier. They opined that the academic research, if any, is of no industrial value. Another inquiry was on the utilisation of a fund named as Central Research Fund (CRF), accumulated by the pharmaceutical industry, to DRAP to promote industrial research. All the respondents believed that, so far, the CRF has not been utilised, although it could be utilised in various ways for promoting the pharmaceutical industry.

#### 2.6.5. Theme 5: ways to improve export potential (facilitators)

Over a question on the ways for the export boost exports, multiple opinions were expressed by the respondents. Almost all respondents believed that the export potential is huge in the Pakistani pharmaceutical industry, and could be improved by adopting the following:

A. Export startup programme with regulatory assistance

Six respondents viewed no need to launch an export startup programme, as a pharmaceutical export is in place, and measures should be taken to improve it. Two respondents were unresponsive to this plan, while 4 out of 12 favoured the launching of an export startup programme with regulatory assistance.

B. Compliance with regulatory standards

All the respondents were of the view that in the local industry, there is no or very little compliance with the cGMPs. Although guidelines exist, yet lack of implementation is the core issue. The importance of compliance with regulatory standards and good practices is emphasised repeatedly as a means of enhancing exports. Improvements in manufacturing standards, adherence to cGMPs, and accreditation with international bodies are seen as potential solutions to this challenge.

C. Provision of locally manufactured APIs

Nine of 12 respondents highlighted the need for basic manufacturing for the production of APIs. With basic manufacturing, the production cost is minimised, thereby the industry will have to find a better way to cope with the price-oriented market competition.

D. Initiation of positive marketing and media campaigns

Positive marketing and media campaigns were suggested as strategies to enhance the image of the domestic pharmaceutical industry, gain trust in the international markets, and improve export potential by 3 out of 12 respondents. They said that the growth potential of pharmaceuticals is there, but unfortunately, it is not being handled positively, as the negative media reports have spoiled the image of the pharmaceutical market.

E. Training of the DRAP officers and industrial staff:

Periodic training of the regulators and industrial staff in collaboration with the international bodies can bring positive change to the current scenario of the export. All the regulators opined the same and said that the regulatory guidelines are there as per international standards, but the understanding of the drug inspectors for DRAP is not up to the mark, and the same is the case with industrial staff.

F. Implementation of laws and rules

Regarding the formation of new laws and rules to enhance exports, almost all of the respondents believed that the laws and rules already exist; therefore, only the implementation of the existing laws governing quality standards is required. One respondent (R10) said that legislation should be business-friendly.

G. Improving DRAP accreditation, certification, and procedures

Eight out of 12 regulators opined that DRAP needs improvement in terms of accreditation and certification. Qualification and membership of the WHO and PICS, respectively, of the DRAP will open new ways of export, particularly to SRA countries. Two respondents recommended that DRAP should improve its procedures of registration and certification procedures by lowering the time frame. One striking piece of information was provided by a provincial regulator dealing with medicine distribution and control (R7), according to whom, the DRAP should also register the internationally accepted moieties, not registered in Pakistan, such as the biologicals, like erythropoietin, and the monoclonal antibodies, which are mainly imported. Such activity could increase the export of the domestic industry.

H. Support from the Government of Pakistan

Respondents believed that support from the Government of Pakistan is necessary for improving pharmaceutical exports. Two respondents suggested that the government should make plans and strategies for export and also for the utilisation of CRF. Almost all respondents said that the government should reduce taxes on the import of raw materials, which, according to them, would reduce production costs. Three respondents also suggested that the government should focus on the DRAP capacity enhancement, as DRAP lacks sufficient human resources and staff.

## 3. Discussion

This study fills a policy gap in the context of Pakistani pharmaceutical exports with a focus on the regulatory experts’ perceptions. Half of the respondents viewed the local pharmaceutical industry as comparable to that in the developed countries, while the rest opined that the domestic industry lacked cGMP compliance and quality procedure guidelines. Application of cGMP is a high priority not only for enabling pharmaceutical exports, but also production of high-quality pharmaceutical products locally (Fatima et al., 2019). The above shortcomings in complying with cGMP and other regulatory standards in quality testing of the raw materials might lead to compromised standards of generic medications, which could adversely affect their export (Babar et al., 2016). A local study reported the financial, technological advancements, infrastructural, administrative, and technical limitations of the industry as the hurdles for the implementation of cGMP and adherence to the quality standards (Fatima et al., 2019). In our previous work, the experts from the pharmacy industry supported the above perception of the regulators (Mubarak et al., 2024b, 2024c).

A valid comparison of national and international industries, according to the three respondents, was difficult as they believed that the pharmaceutical units were categorised at low, middle, and high levels – the units at middle and high levels could be compared to the industry in the developed countries. All respondents agreed on the existence of the export potential of the national pharmaceutical industry, yet offset by several addressable challenges hindered the transformation of the above potential into a factual export. Against the existence of export potential, the non-adherence of the pharmaceutical industry to cGMPs and other regulatory standards was highlighted again as the biggest export impediment. The possible reasons for non-adherence to the cGMP have already been discussed in the previous text. The pharmaceutical industry uses non-GMP-compliant equipment, contrary to the ICH and FDA guidelines (FDA, 2022), which could be regarded as a regulatory gap.

The investment amount is perceived to be linked with the willingness of the investors to carry out a long-term and quality-oriented business, a necessary factor for export. However, regarding the imposition of a minimum limit for investment by the regulatory authority for business startups, some regulators were negative about DRAP-imposed investment limits, considering it as the discretion of the company. Further, the Ministry of Commerce, Pakistan, does not impose any capital requirements for a business startup (Ministry of Commerce, 2021). Other respondents viewed mandatory imposition of the investment limit as necessary for quality standards and sustainable business growth. A respondent (R9) claimed that the cGMP guidelines had a part of the investment for setting up a business to be considered by a company, although the above information could not be confirmed through the latest version of the cGMPs (DRAP, 2023).

Regarding the mandatory export requirement for a product after its local registration, the regulators opined that it is a company's discretion. Though binding of firms for export by the regulatory authority would accelerate export, it would not be possible for the regulatory body. Thus, an independent governmental body could persuade the firms to export their specific generics, at least those that are leading and successful in the local market.

A priority registration of the next molecule of the same company after registration for an export product has been revealed by a couple of respondents as the only incentive for the company to export its products. Contrary to the above, the Indian government has implemented multiple initiatives, such as production-linked incentives, key starting materials, drug intermediates, and active pharmaceutical ingredients schemes, to catalyze growth in the pharmaceutical sector and domestic production of bulk drugs. As a result, financial incentives were offered for 41 products over 6 years. Fermentation products received 20%, 15%, and 5% incentives, respectively, for the first 4 years, the next year, and for the final year. Chemical synthetic products receive a 10% incentive for 6 years for companies meeting the investment thresholds and eligibility criteria. The scheme costs around 69.40 billion Indian rupees. The promotion of bulk drug parks is another initiative whereby financial aid is for creating common infrastructure facilities in three selected bulk drug parks provided under the scheme, with up to 90% assistance in northeastern and hilly areas and 70% in other states (MakeinIndia, 2020). Long-term growth was also proposed through the establishment of API megapacks (Bhutta, 2022). Pakistan also started a similar scheme to that of India for self-reliance in the API manufacturing industry as short-term incentives, including tax holidays, zero customs duty, and financial aid.

In Pakistan, DRAP imposes control over drug prices, whereby the companies cannot fix the price for a product by themself (Ahmad & Bukhari, 2024). The regulators expressed the non-existence of incentives in terms of a greater price or enhanced profit margins for the firms involved in exports, except one respondent, according to which the local price control was exempted, and the export product manufacturer could set the export price. The drug price policy of 2018 confirmed the above statement, according to which domestic products with export approval from the developed nations or the WHO are excluded from price regulation in the local market as long as the free-on-board price is higher than the ex-factory price in the local market. The above is to promote the production and export of high-quality drugs (DRAP, 2018a).

An unlimited relaxation of the regulatory requirement of BE testing for registration of generics and the absence of any incentives for the above were considered barriers to the performance of BE studies. While the countries with SRA impose BE and other certifications from qualified agencies (FDA, 2002). Thus, despite the potential, national pharmaceutical companies could not export to such countries. Unavailability of accredited BE testing facilities in Pakistan, firms are forced to get BE studies from other countries, which incurs significant expenses. BE studies from abroad are further hampered due to the involvement of foreign exchange and money transfers (Hasan, 2012), which is not legitimate in Pakistan, according to the current laws. The above situation is being improved with the establishment of DRAP-licensed bioequivalence centres (DRAP, 2022d). Currently, there are altogether six BE centres, inclusive of a state-owned BE testing Lab, Pakistan Drug Testing & Research Centre, Lahore. However, none of the above are accredited by any international bodies. The performance of these centres is unclear for export facilitation, and could not be linked with the regulatory requirement of BE testing for generic registration or meeting international requirements. Earlier, two BE centres were discontinued, probably due to a lack of clientele.

To address the issues, regulators suggested government facilitation for the industry in the performance of BE studies. Certain other areas were also identified for improvement to cope with the international market competition. Development of industrial research and development (R&D) was the most emphasised area, which was almost non-existent in the domestic industry, wherein mostly the generics are prepared. A study suggested prioritisation of industrial R&D to expand global market share. Nevertheless, developing a new moiety is challenging as it requires tremendous spending, considerable time, and successful passing of the clinical trials (Healthaffairs, 2006). National pharmaceutical companies lack a budget for R&D and do not have a single R&D laboratory, in contrast to their Indian counterpart, which have allocated 8-11% of revenue for R&D (Jibran, 2015).

The academia-industry linkage (AIL) is also a missing area – the industry-tailored academic research could benefit the industry in terms of boosting R&D and, thereby, business growth. One of the respondents (R7) suggested that DRAP should bridge AIL and that the faculty should be recruited from industry and regulatory sectors to teach effectively practice-oriented concepts. The AIL and compliance with R&D standards could be improved by strategic utilisation of the central research fund (CRF). The CRF is being deposited to DRAP as 1% of the profit of the industrial annual sales before tax (DRAP, 2018b). For long, this huge amount has remained unutilised. This fund could be utilised for capacity building of the regulatory sector, establishing the R&D laboratories by soft loaning to the small firms, facilitation in BE studies, and academia-industrial collaborative research projects. It has been suggested to establish ten Food and Drug Administration-accredited export-dedicated drug manufacturing units. The Joint Trade Development Authority of Pakistan (TDAP), DRAP, Foreign Affairs, Chambers of Commerce, and other relevant authorities could initiate a one-window operation for the issuance of the required drug export documents (Jibran, 2015).

The domestic pharmaceutical industry encounters overarching issues, especially in labour productivity, electricity supply, and costs relative to peer countries, such as China, Malaysia, and India (Khan et al., 2021). All of the above lead to costlier manufacturing. Very few (only three) domestic pharmaceutical manufacturers have the WHO prequalification certification, which they have achieved at significant individual cost, without any government enforcement, facilitation, or financial support (Khan et al., 2021).

Among other factors, such as the cGMP compliance, regarded as the prime factor in boosting pharmaceutical exports by the respondents, the others were providing the incentive and facilitation for the local API manufacturing, which, according to one respondent (R7), could be made possible by the utilisation of CRF. The capacity to generate the most common salts or their precursor could benefit the industry in terms of costs.

The trust of importing countries in domestic products was seen as essential for export, which could be built through compliance with regulatory bodies, high-quality products, and improved marketing standards. A positive image of the domestic pharmaceutical industry through media campaigns and promotional activities was also expressed by the respondents. In the past, the media reported certain incidents of counterfeit medicines, of which, the one in the Punjab Institute of Cardiology Lahore, claiming 100 deaths of heart patients due to adulterated and counterfeit medicines, was on the top (Chaudhry, 2013). A report ‘Why our drug markets askew,’ published in the daily DAWN (a renowned English newspaper), claimed that most drugs in Pakistan were fake/counterfeit (Attarwala, 2021). Such media reports portray a negative global image of national pharmaceuticals that reduces the trust of the regulatory bodies in developed countries and results in reduced sales of domestic products in global markets. Recently, there have been five WHO-prequalified drug testing laboratories, relative to only two in 2020 (WHO, 2023). This number is greater than any other Asian country presently, thus the image of the domestic regulatory system and the product quality is envisioned to improve internationally.

DRAP was started to recruit and develop skilled professionals, modernise the system, set up pharmacovigilance, upgrade drug testing laboratories and human resources, and equipment (Atif et al., 2017; Jamshed et al., 2015). Respondents stressed the need for the training of the DRAP human resources on the regulatory measures for export and trends in the international markets. Reinforcing infrastructure and human resource development are the major ways to execute regulatory reforms. The credibility and efficiency of the DRAP can be enhanced by establishing external linkages and accreditations, as reported (Jamshed et al., 2015; Zaidi et al., 2015) with regulatory agencies of other countries, particularly of FDA, European Medicines Agency (EMA), and others. A need for such regulatory collaborations has already been emphasised to ensure public health and timely patient access to treatments. Currently, developing shared approaches and standards while maintaining independence under respective legal mandates is the focus of regulators from different countries. In this connection, since 2004, EMA, FDA, and other authorities have been engaged in strategic partnerships across regions through a series of meetings called clusters, aimed at providing a platform for experts and sharing experiences and insights on various areas of regulatory science (Teixeira et al., 2020). Further DRAP should focus on getting certified from PICS and WHO to increase access to bigger stringent markets. Nevertheless, the main issue faced by DRAP in enforcing the above is the budgetary allocation (Jamshed et al., 2015), which again could be resolved by the appropriate utilisation of the CRF.

DRAP has two specialised sections to deal with anticancer and biologicals. The areas of DRAP requiring improvement could be visualised from its comparison to any SRA country. Like SRA, ideally, the regulatory system should be divided into main divisions with sections carrying the appropriate number and quality of human resources. It must have separate structural designs, for the product specialisation, based on: (a) products’ clinical categories and patient populations, and (b) research to deals with pharmacoepidemiology, pharmacogenomics, biostatistics, special pharmacology, and specialised neuropsychopharmacology (Rasheed et al., 2019). One respondent (R4) suggested that the government should also support exports by providing export facilitation zones, reducing taxes and customs duties on APIs, and giving reasonable product prices and profit margins on medicines by subsidising labour costs, electricity charges, and eliminating the regulatory limitations as discussed above.

## 4. Limitations of the study

Such types of studies are usually limited by personal design biases. However, in this study, the above limitation was attempted to be mitigated by encouraging the respondents to express themselves without any interruption. The probing questions were asked only to clarify the aspects under discussion without any additions by the interviewer. Further, no alteration was considered in coding if any respondent changed their previous response based on the next question, as indicated previously. The participants included in this study were mainly from Lahore and Islamabad due to convenience. The participants from other provinces, except a few (2 only) from Balochistan, could not be included due to unresponsive calls, which could cause variations in the findings of the present study. Nevertheless, such shortcomings are inherent in these types of studies (Hashmi et al., 2017). The smaller number of respondents and their belonging to the same organisation might be challenging for establishing the validity of the findings, yet the focus of this study was to investigate the perception of the regulatory experts. Thus, the findings could be generalisable with caution. The sample size was confined to only 12 experts. This could be another limitation, and, in the future, the study can be expanded with a greater number of participants and also from the remaining regional regulatory offices. The findings of the study showed that, being a major stakeholder, the point of view of the pharmaceutical industry on this area should also be taken.

## 5. Conclusion

In 5 themes and 20 categories/codes, the existence of export potential was highlighted, which had been hampered due to inadequate industrial research and development, non-adherence with the current standards of good manufacturing practices, lack of regulatory requirements for high-tech equipment, non-existent academia-industry collaboration, and a minor locally manufactured active pharmaceutical ingredient. Other barriers were the lack of support from the government and accreditation with international organisations. The Drug Regulatory Authority of Pakistan could play its role in conjunction with the Trade Development Authority of Pakistan in enhancing pharmaceutical exports. Few measures are required to tap the maximum advantage of the export potential. There is a serious need for developing research and development for the growth of the pharmaceutical sector. Academia-industrial collaboration, with regulatory assistance, is required to enhance research and development. International marketing campaigns and expos for the launch of generic products should be arranged, and the growth of the pharmaceutical industry should be emphasised. The image of the pharmaceutical industry should be enhanced, and the negative campaigns should be discouraged. Government support to DRAP in terms of surprise audits and visits to the industry can help ensure compliance with the set standards. The international market share of the domestic pharmaceutical industry to SRA could be raised by mitigating the above challenges or adopting certain measures stated above.

### 5.1. Recommendations

#### 5.1.1. Recommendations for industry

- The industry should improve itself in terms of quality control and compliance with cGMP standards.
- The R&D-based projects are required to be started immediately through AILs.
- The industry should start the manufacturing of active, raw materials, and excipients locally to reduce the manufacturing costs, a competitive edge for the national industry in terms of production cost. It would be more profitable to spend on a better share of exports.

#### 5.1.2. Recommendations for DRAP

- CRF deposited to DRAP by industries should be utilised to boost R&D and capacity building of DRAP itself in terms of human resources through training.
- DRAP should focus on getting registration from the Pharmaceutical Inspection Convention (PICS) and the WHO to facilitate the covering of the gap between PICS-approved countries for export. This is expected to gain access to stringent markets.
- DRAP should ensure that the cGMP guidelines are being observed in industry through the strict monitoring and inspections

#### 5.1.3. Recommendations for government

- The government should arrange a cessation of the unrealistic negative campaigns on spurious and counterfeit medicines to save the image of the Pakistani industry.
- The government should support the start of the basic manufacturing of active and raw materials locally to reduce the material and manufacturing costs, mandatory for a better share of exports.
- The government should offer incentives, such as the tax rebate, and allocation of reasonable product prices for the companies involved in the research and development.
- The government should provide subsidised units of electricity to the industries reducing labour costs and should relax the policies of export for pharmaceuticals.
- Govt should ensure the proper functioning of the export promotion committee. All the tasks assigned to the committee should be monitored properly to achieve the desired outcome.

#### 5.1.4. Recommendations for academia

- The academia should announce research-based projects in conjunction with DRAP and the Pharmacy Council of Pakistan.
- Academia should hire human resources from regulatory agencies and industries for practice-based knowledge sharing with students.

## Author contribution

Conceptualisation: FKH, NA, NIB. Data curation: ZM, MM, FA, FKH, NA. Formal analysis: ZM, AJ, FBB. Investigation: ZM, MM, AR, FA, HS. Methodology: ZM, FBB, GDR, NIB. Project administration: ZIB. Supervision: NIB. Visualisation: GKH, NA, NIB. Writing original draft: ZM, MM, HS, GDR. Writing, reviewing and editing: ZM, AR, FA, FKH, NA.

## Supplementary Material

Interview Guide.docx

## Data Availability

The original contributions described in this study are included in the article. Further inquiries can be directed to the corresponding author.
