# Supplementary material for: Barriers to and enablers of Pakistani pharmaceutical export to regulated markets: regulatory perspective
Source: J Pharm Policy Pract. 2025 Nov 28;18(1):2564828. doi: 10.1080/20523211.2025.2564828 (PMC12667322; doi:10.1080/20523211.2025.2564828)
Supplement: Interview Guide.docx [file JPPP_A_2564828_SM6847.docx]

**Interview Guide**

(Regulator Part)

| **1** | **Name code of the person** |  |
| --- | --- | --- |
| **2** | **Gender** |  |
| **3** | **Designation** |  |
| **4** | **Years of experience** |  |
| **5** | **Qualification** |  |
| **6** | **Age** |  |

1. How the national pharmaceutical industry is compared to the Pharma Industry in the developed countries?

----------------------------------------------------------------------------------------------------------------------------------------------------------------------------------------------------------------------------------

1. Should there be a limit of the minimum investment for startup of business?

----------------------------------------------------------------------------------------------------------------------------------------------------------------------------------------------------------------------------------

1. Has the National pharmaceutical Industry export potential? How export potential can be improved?

----------------------------------------------------------------------------------------------------------------

----------------------------------------------------------------------------------------------------------------------------------------------------------------------------------------------------------------------------------

1. Is there any regulatory requirement to install high-Tech instruments in industry to obtain the manufacturing license?

---------------------------------------------------------------------------------------------------------------------------------------------------------------------------------------------------------------------------------

1. Is there any regulatory requirement to apply for the export registrations after obtaining the manufacturing license and registration for the local market?

----------------------------------------------------------------------------------------------------------------------------------------------------------------------------------------------------------------------------------

1. Is there any incentive for local pharmaceutical industry regarding grant of local drug registrations who already export their products?

----------------------------------------------------------------------------------------------------------------------------------------------------------------------------------------------------------------------------------

1. Is there any incentive for pharmaceutical industries regarding the price or profit margins who already export their pharmaceutical products?

----------------------------------------------------------------------------------------------------------------------------------------------------------------------------------------------------------------------------------

1. Is there any incentive for pharmaceutical industry if they want to carry out bioequivalence studies?

----------------------------------------------------------------------------------------------------------------------------------------------------------------------------------------------------------------------------------

1. What is the view of the regulatory authority on the research and development of a new drug molecule in local Research and Development activities?

----------------------------------------------------------------------------------------------------------------------------------------------------------------------------------------------------------------------------------

1. Is regulatory legislation required in improving local production of pharmaceuticals that match the international standards?

----------------------------------------------------------------------------------------------------------------------------------------------------------------------------------------------------------------------------------

1. What is the value of local Research carried out in academia on the time-tested drugs?

----------------------------------------------------------------------------------------------------------------------------------------------------------------------------------------------------------------------------------

1. Will the incentives given in Question 8, 9 and 10 benefit the local pharmaceutical industry in terms of export?

**----------------------------------------------------------------------------------------------------------------------------------------------------------------------------------------------------------------------------------**

1. What is the role of the Drug Regulatory Authority of Pakistan (DRAP) in the Academia-Industrial Collaboration?

---------------------------------------------------------------------------------------------------------------------------------------------------------------------------------------------------------------------------------

1. What can be the affective utility of the fund submitted, by pharmaceutical industry to DRAP in lieu of research and development?

----------------------------------------------------------------------------------------------------------------------------------------------------------------------------------------------------------------------------------

1. What is the proposal to boost pharmaceutical export? What role DRAP can play in it?

----------------------------------------------------------------------------------------------------------------------------------------------------------------------------------------------------------------------------------

1. What are your views about any export start-up program? What role DRAP can play in the export start-up program?

----------------------------------------------------------------------------------------------------------------------------------------------------------------------------------------------------------------------------------

1. Do the expenditures in power backups are covered in the pricing of the pharmaceutical products?

----------------------------------------------------------------------------------------------------------------------------------------------------------------------------------------------------------------------------------

1. Do you provide any training to the local pharmaceutical industry for skill development or any other technical aspect for the development of the pharmaceutical sector?

---------------------------------------------------------------------------------------------------------------------------------------------------------------------------------------------------------------------------------
